# Progressive Line Processing of Kernel RX Anomaly Detection Algorithm for Hyperspectral Imagery

**DOI:** 10.3390/s17081815

**Published:** 2017-08-07

**Authors:** Chunhui Zhao, Weiwei Deng, Yiming Yan, Xifeng Yao

**Affiliations:** College of Information and Communication Engineering, Harbin Engineering University, Harbin 150001, China; Dengweiwei@hrbeu.edu.cn (W.D.); yanyiming@hrbeu.edu.cn (Y.Y.); xf.yao1020@gmail.com (X.Y.)

**Keywords:** hyperspectral imagery, KRX anomaly detection, real-time algorithm, progressive line processing, the causal sliding window

## Abstract

The Kernel-RX detector (KRXD) has attracted widespread interest in hyperspectral image processing with the utilization of nonlinear information. However, the kernelization of hyperspectral data leads to poor execution efficiency in KRXD. This paper presents an approach to the progressive line processing of KRXD (PLP-KRXD) that can perform KRXD line by line (the main data acquisition pattern). Parallel causal sliding windows are defined to ensure the causality of PLP-KRXD. Then, with the employment of the Woodbury matrix identity and the matrix inversion lemma, PLP-KRXD has the capacity to recursively update the kernel matrices, thereby avoiding a great many repetitive calculations of complex matrices, and greatly reducing the algorithm’s complexity. To substantiate the usefulness and effectiveness of PLP-KRXD, three groups of hyperspectral datasets are used to conduct experiments.

## 1. Introduction

Because of abundant spectral information, hyperspectral imagery (HSI) has the potential to discover the subtle differences of ground materials that cannot be visually inspected in a multispectral image [1]. As such, it has gained widespread attention in the fields of geology, the military, the mining industry, and medical imaging, among others [2,3,4]. Anomaly detection, which is one of the most popular branches in hyperspectral image processing, is capable of uncovering many masked targets of interest without a priori spectral knowledge, and as such, it generally conforms to practical conditions, and has gradually been considered very effective and useful in HSI [5,6,7,8].

The Reed–Xiaoli detector (RXD) [9] is a widely used anomaly detection algorithm, and is regarded as a benchmark algorithm in HSI [10,11,12]. Derived from the generalized likelihood ratio test (CFAR), it finds abnormal points by calculating the Mahalanobis distance between the background and the pixel under test (PUT) in a multivariate Gaussian background. However, the RXD algorithm merely utilizes the low-order linear statistics of hyperspectral data, thus leading to inferior detection accuracy in complex and changeable ground distributions. To address this issue, kernel-RXD (KRXD), a kernel version of the RXD algorithm, is presented by Kwon and Nasrabadi [13]. KRXD exhaustively mines the high-order correlation between spectral bands via a kernel function. It can vastly improve the detection accuracy as compared to the RXD when original data are mixed in a non-linear model, which is always the case. Unfortunately, the data kernelization in KRXD produces lots of multiplications and the inversion of matrices, thereby consuming a lot of processing time.

Recently, real-time anomaly detection has gained wide attention in HSI [14,15,16,17,18,19,20,21]. It is particularly significant, as some moving objects are highly desirable to be detected on a timely basis. Furthermore, with the huge pressure of big data storage, it becomes the inevitable trend to perform anomaly detection in real time. It is worth noting that the realization of real-time processing is mainly dependent on the three data acquisition formats [17,18].

The first such format is the band interleaved pixel (BIP) format, which performs anomaly detection pixel by pixel. Two real-time algorithms based on the RXD, the global real-time causal RX detector (GRTC-RXD), and the local real-time causal RX detector (LRTC-RXD), are discussed in [19,20] in terms of BIP, respectively. Both real-time algorithms solve the problem of causality (the one requirement of real-time processing), and only the data samples already visited can be used to detect PUT; no future data samples should be involved in the data processing [21]. They also enhance the execution efficiency by recursively updating the covariance matrices via the Woodbury matrix identity [22]. While the GRTC-RXD utilizes all processed data before the PUT as the background, LRTC-RXD regards the data contained in sliding causal array windows as the background to perform real-time detection. As these algorithms usually have an undesirable detection output by using the low-order statistics of the HSI dataset, a real-time algorithm based on the KRXD to yield better detection accuracy with much shorter processing time has been recently proposed [23]. The second such format is the band sequential (BSQ) format, which processes data samples band by band. According to the BSQ format, progressive band processing anomaly detection (PBP-AD) could be implemented band by band progressively while the data acquisition is still ongoing [24]. The third and final such format is the band interleaved line (BIL) format, which collects data line by line. Since acquiring data with a push broom scanner has gradually become the mainstream for hyperspectral imaging sensors [25,26], the literature [26] presents a real-time anomaly detector based on the BIL format. By using a linear algebra-based strategy, it effectively updates the inverse covariance, thus avoiding complex computation. The renewal process of correlation matrices only separately considers the data going in and out of the dual window. Yet, it does not re-derive the covariance matrix by treating these data and the duplicate data as a whole, leading to the loss of some information in recursion. Additionally, the low detection accuracy is still a challenge, as it is based on the RXD.

Therefore, to tackle the aforementioned issues, this paper presents an approach to the progressive line processing of KRXD (PLP-KRXD) via BIL. To ensure the causality of the detection system, a new type of local window (parallel causal sliding window) is defined. In this case, hyperspectral data can be collected line by line progressively during the process of line acquisition. As the proposed method is updated recursively using the Woodbury matrix identity and the matrix inversion lemma [27], it avoids having to recalculate the previously processed data lines, thereby significantly reducing computational complexity. Experimental results demonstrate that the PLP-KRXD keeps the detection accuracy unchanged and speeds up the execution efficiency compared to the KRXD.

## 2. Methods

### 2.1. KRX Anomaly Detector

In this section, we review the KRXD, a nonlinear version of the RXD, which ideally can separate the background and PUT by sufficiently utilizing high-order statistics. Suppose that each data sample vector consists of J spectral bands, denoted by $x_{i}={\left[x_{i1},x_{i2},\cdots x_{iJ}\right]}^{T}$. The KRXD maps the original data $X_{B}=\left[x_{1},x_{2},\cdots ,x_{M}\right]$, where M is the number of the data sample vectors, to the high-dimensional feature space with the nonlinear function as follows

(1) $$\Phi \left(X_{B}\right)=\Phi \left(X_{B}\right)=\left[\Phi \left(x_{1}\right),\Phi \left(x_{2}\right),\cdots \Phi \left(x_{M}\right)\right]$$

where Φ(⋅) is the kernel function. With the same assumption as for the RXD, the KRXD now consists of two Gaussian distributions, thereby modeling the two hypotheses as

(2) $$\{\begin{array}{l} H_{0\Phi }:\Phi \left(x\right)=n_{\Phi }\;(\mathrm{Target}\;\mathrm{absent})\\ H_{1\Phi }:\Phi \left(x\right)=a_{\Phi }\Phi \left(s\right)+n_{\Phi }\;(\mathrm{Target}\;\mathrm{present}) \end{array}$$

where $a_{\Phi }=0$ under $H_{0\Phi }$ and $a_{\Phi }>0$ under $H_{1\Phi }$, respectively. Φ(s) and $n_{\Phi }$ separately represent the spectral signal and background clutter in the feature space. Then, the corresponding representative form of the KRXD is compactly given by

(3) $$\delta^{KRXD}\left(\Phi \left(r\right)\right)={\left(\Phi \left(r\right)-{\hat{\mu }}_{B\Phi }\right)}^{T}{\hat{K}}_{B\Phi }^{-1}\left(\Phi \left(r\right)-{\hat{\mu }}_{B\Phi }\right)$$

where r is the PUT, and ${\hat{\mu }}_{B\Phi }$ and ${\hat{K}}_{B\Phi }$ are the estimated mean and covariance matrix of the background data in the feature space, respectively. According to the specific kernelization and derivation, we have

(4) $$k_{r}^{T}=k\left(r,X_{B}\right)-\left(\frac{1}{M}\sum_{i=1}^{M}k\left(r,x_{i}\right)\right)1_{1\times M}$$

(5) $$k_{\hat{\mu }}^{T}=\left(\frac{1}{M}\sum_{i=1}^{M}k\left(x_{i},X_{B}\right)\right)-\left(\frac{1}{M^{2}}\sum_{i=1}^{M}\sum_{j=1}^{M}k\left(x_{i},x_{j}\right)\right)1_{1\times M}$$

where $1_{1\times M}$ is an 1×M vector with all values equal to 1. The Gram matrix is expressed by $K_{B}=k\left(X_{B},X_{B}\right)$. So, the final KRXD algorithm can be simplified as

(6) $$\delta^{KRXD}\left(\Phi \left(r\right)\right)={\left(k_{r}^{T}-k_{\mu }^{T}\right)}^{T}K_{B}^{-1}\left(k_{r}^{T}-k_{\mu }^{T}\right).$$

Choosing the proper kernel function could exert a crucial influence on the performance of the kernel-based learning algorithm. The polynomial kernel function implements the dot product in feature space with an effective kernel trick, expressed as follows:

(7) $$k\left(x,x_{i}\right)={\left(x^{T}x_{i}\right)}^{d},d\in N$$

where d is a positive constant.

### 2.2. Proposed Progressive Line Processing of the KRX Anomaly Detector

Though the KRXD has desirable detection accuracy, it is excessively time-consuming due to the computation of complex matrices. Fortunately, the implementation of the KRXD can be improved to perform in real-time. Interestingly, the issue of performing KRXD with current hyperspectral imaging sensors working in a push-broom fashion has not received much attention in the past. Therefore, the idea is presented that the PLP-KRXD arises from a need to implement the KRXD in real time using a new type of a causal sliding window.

#### 2.2.1. Push Broom Scanner

**Figure 1:**
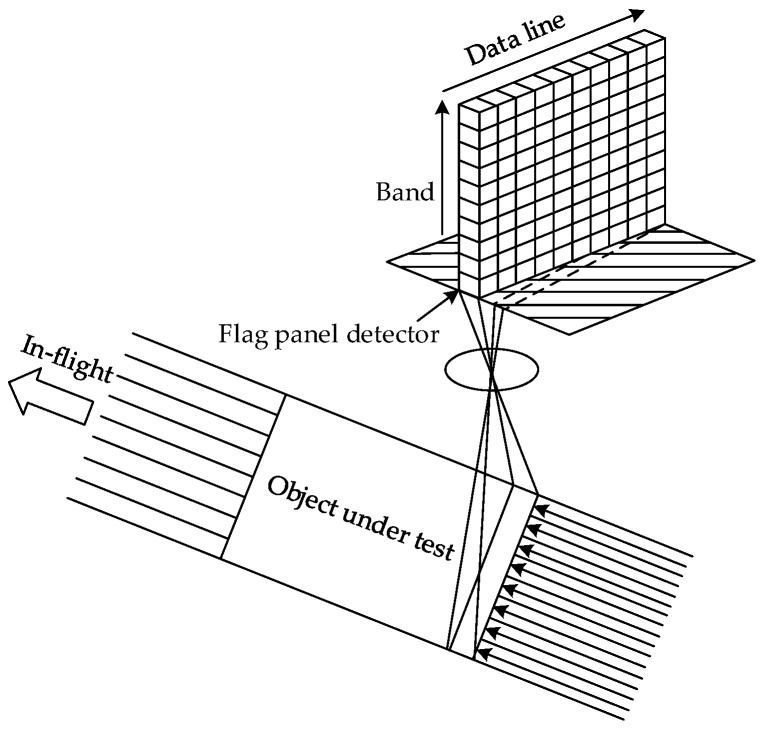
The imaging principle of push broom scanner.

A push broom scanner or a so-called along-track scanner is a device for obtaining images with spectroscopic sensors. Figure 1 shows the imaging principle of a push broom scanner. It employs a flat panel detector to simultaneously collect all data as spectral information in a line-by-line fashion. Meanwhile, spatial imaging is attained as the platform moves along with the flight direction. A push broom scanner can achieve high system sensitivity and a hight signal-to-noise ratio (SNR), because it gazes at a particular area for a long time.

#### 2.2.2. Parallel Causal Sliding Windows

**Figure 2:**
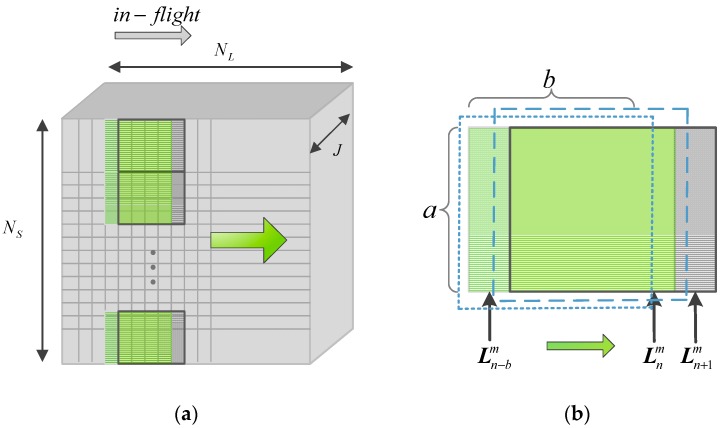
Schematic representation of data acquisition and processing: (**a**) The process of the parallel causal sliding windows; (**b**) The causal sliding rectangle window at $L_{n}^{m}$ and $L_{n+1}^{m}$.

Hyperspectral anomaly detection adopts the traditional double-window model to detect targets of interest in most cases. However, causality, which is the prerequisite of real-time detection, cannot be attained. This is because the dual window must process the future data sample vectors after the PUT, which leads to the non-causality of the processing system. To address this issue, a new type of parallel causal sliding window based on progressive line processing is defined, as shown in Figure 2. As is evident from Figure 2a, the implementation is attained by a series of causal sliding rectangle windows in a parallel alignment. In this manner, each data line can be divided into several parts and simultaneously detected, and the data can be progressively processed line by line. To further illustrate the processing of this algorithm, Figure 2b shows a representation of line-by-line real-time processing in the cross-track direction. Let $L_{n}^{m}$ denote the $n^{th}$ data processing line in the $m^{th}$ causal sliding rectangle window specified by its length a and width b. It should be noted that the currently processed line is not contained in the causal sliding rectangle window. When the causal sliding rectangle window slides from $L_{n}^{m}$ (the dotted line window) to $L_{n+1}^{m}$ (the dashed line window), most of the data line vectors collected do not vary, except for the contribution of the data line vectors leaving and entering the causal sliding rectangle window ($L_{n-b}^{m}$ and $L_{n+1}^{m}$).

#### 2.2.3. Causal KRXD Algorithm

Now we assume that L(n) makes up of all the data lines up to $L_{n-1}^{m}$, i.e., $L\left(n\right)=\cup_{i=n-b}^{n-1}L_{i}^{m}$, where b is equal to the number of lines contained in the current causal sliding rectangle window, and $L_{i}^{m}$ is the $i^{th}$ data line. As a result, the KRXD can be causally designed as follows:

(8) $$\delta^{KRXD}\left(\Phi \left(L_{n}^{m}\right)\right)={\left(k_{L_{n}^{m}}^{T}-k_{\mu }^{T}\left(L\left(n\right)\right)\right)}^{T}K_{B}^{-1}\left(L\left(n\right)\right)\left(k_{L_{n}^{m}}^{T}-k_{\mu }^{T}\left(L\left(n\right)\right)\right)$$

where $K_{B}\left(L\left(n\right)\right)=k\left(X_{B}\left(L\left(n\right)\right),X_{B}\left(L\left(n\right)\right)\right)$ is the causal Gram matrix, and $X_{B}\left(L\left(n\right)\right)$ is the causal background matrix formed by all of the data line vectors in the current causal sliding rectangle window.

In order to satisfy the causality, $k_{\mu }^{T}\left(L\left(n\right)\right)$ and $k_{L_{n}^{m}}^{T}$ must be specified by:

(9) $$k_{\mu }^{T}\left(L\left(n\right)\right)=\frac{1}{b}\sum_{i=1}^{b}k\left(L_{n}^{m},X_{B}\left(L\left(n\right)\right)\right)-\frac{1}{b^{2}}\sum_{i=1}^{b}\sum_{j=1}^{b}k\left(L_{i}^{m},L_{j}^{m}\right)1_{b\times w}$$

(10) $$k_{L_{n}^{m}}^{T}\left(L\left(n\right)\right)=k\left(L_{n}^{m},X_{B}\left(L\left(n\right)\right)\right)-\frac{1}{b}\sum_{i=1}^{b}k\left(L_{n}^{m},L_{i}^{m}\right)1_{b\times w}$$

where w is the total number of entire data sample vectors in a causal sliding rectangle window.

#### 2.2.4. Progressive Line Processing of the KRX Anomaly Detector

Theoretically, according to Equation (8), the causality of KRXD is ensured so that it can be implemented in real-time. However, recalculating $K_{B}^{-1}\left(L\left(n\right)\right)$ is excessively time consuming as long as the causal sliding window moves. This processing time scarcely meets the time restraint for real-time implementation. Therefore, in what follows, we derive the recursive update equation of $K_{B}^{-1}\left(L\left(n\right)\right)$ to solve this issue.

Let $X_{B}\left(L\left(n\right)\right)=\left(L_{n-b}^{m},L_{n-b+1}^{m},\cdots L_{n-1}^{m}\right)$ be the background data sample vectors in the current causal sliding rectangle window at $L_{n}^{m}$. Using the polynomial kernel function in Equation (7), $K_{B}\left(L\left(n\right)\right)$ can be given by

(11) $$\begin{array}{l} K_{B}\left(L\left(n\right)\right)=k\left(X_{B}\left(L\left(n\right)\right),X_{B}\left(L\left(n\right)\right)\right)={\left(X_{B}{\left(L\left(n\right)\right)}^{T}X_{B}\left(L\left(n\right)\right)\right)}^{d}\\ =\left[\begin{array}{cccc} k\left(L_{n-b}^{m},L_{n-b}^{m}\right) & k\left(L_{n-b}^{m},L_{n-b+1}^{m}\right) & \cdots & k\left(L_{n-b}^{m},L_{n-1}^{m}\right)\\ k\left(L_{n-b+1}^{m},L_{n-b}^{m}\right) & k\left(L_{n-b+1}^{m},L_{n-b+1}^{m}\right) & \cdots & k\left(L_{n-b+1}^{m},L_{n-1}^{m}\right)\\ \vdots & \vdots & \ddots & \vdots \\ k\left(L_{n-1}^{m},L_{n-b}^{m}\right) & k\left(L_{n-1}^{m},L_{n-b+1}^{m}\right) & \cdots & k\left(L_{n-1}^{m},L_{n-1}^{m}\right) \end{array}\right]=\left[\begin{array}{cc} \rho_{L_{n}^{m}} & \alpha_{L_{n}^{m}}\\ \alpha_{L_{n}^{n}}^{T} & K_{bw} \end{array}\right] \end{array}$$

where $\alpha_{L_{n}^{m}}$ and $\rho_{L_{n}^{m}}$ are separately defined by $\alpha_{L_{n}^{m}}=\left[k\left(L_{n-b}^{m},L_{n-b+1}^{m}\right),k\left(L_{n-b}^{m},L_{n-b+2}^{m}\right),\cdots k\left(L_{n-b}^{m},L_{n-1}^{m}\right)\right]$, and $\rho_{L_{n}^{m}}=k\left(L_{n-b}^{m},L_{n-b}^{m}\right)$. Taking the advantage of the matrix inversion lemma, $K_{B}^{-1}\left(L\left(n\right)\right)$ can be expressed as:

(12) $$\begin{array}{l} K_{B}^{-1}\left(L\left(n\right)\right)={\left[\begin{array}{cc} \rho_{L_{n}^{m}} & \alpha_{L_{n}^{m}}\\ \alpha_{L_{n}^{n}}^{T} & K_{bw} \end{array}\right]}^{-1}=\left[\begin{array}{cc} \rho_{L_{n}^{m}}^{-1}+\rho_{L_{n}^{m}}^{-1}\alpha_{L_{n}^{m}}\kappa^{-1}\alpha_{L_{n}^{n}}^{T}\rho^{-1} & -\rho_{L_{n}^{m}}^{-1}\alpha_{L_{n}^{m}}\kappa^{-1}\\ -\kappa^{-1}\alpha_{L_{n}^{n}}^{T}\rho_{L_{n}^{m}}^{-1} & \kappa^{-1} \end{array}\right]\\ \kappa =K_{bw}-\alpha_{L_{n}^{n}}^{T}\rho_{L_{n}^{m}}^{-1}\alpha_{L_{n}^{m}} \end{array}.$$

Note that $\kappa^{-1}$ can be acquired from $K_{B}^{-1}\left(L\left(n\right)\right)$. As follows from Equation (12), we can obtain

(13) $$K_{bw}^{-1}={\left(\kappa +\alpha_{L_{n}^{n}}^{T}\rho_{L_{n}^{m}}^{-1}\alpha_{L_{n}^{m}}\right)}^{-1}.$$

For the purpose of finding a recursive formula of $K_{bw}^{-1}$ to reduce the computational complexity, we evaluate the Woodbury matrix identity expressing $K_{bw}^{-1}$:

(14) $$K_{bw}^{-1}={\left(\kappa +\alpha_{L_{n}^{n}}^{T}\rho_{L_{n}^{m}}^{-1}\alpha_{L_{n}^{m}}\right)}^{-1}=\kappa^{-1}-\kappa^{-1}\alpha_{L_{n}^{n}}^{T}{\left(\rho_{L_{n}^{m}}+\alpha_{L_{n}^{m}}\kappa^{-1}\alpha_{L_{n}^{n}}^{T}\right)}^{-1}\alpha_{L_{n}^{m}}\kappa^{-1}.$$

Similarly, the kernel Gram matrix at the ${\left(n+1\right)}^{th}$data processing line in the $m^{th}$ causal sliding rectangle window at $L_{n+1}^{m}$ can be calculated as

(15) $$\begin{array}{l} K_{B}\left(L\left(n+1\right)\right)=k\left(X_{B}\left(L\left(n+1\right)\right),X_{B}\left(L\left(n+1\right)\right)\right)={\left(X_{B}{\left(L\left(n+1\right)\right)}^{T}X_{B}\left(L\left(n+1\right)\right)\right)}^{d}\\ =\left[\begin{array}{cccc} k\left(L_{n-b+1}^{m},L_{n-b+1}^{m}\right) & \cdots & k\left(L_{n-b+1}^{m},L_{n-1}^{m}\right) & k\left(L_{n-b+1}^{m},L_{n}^{m}\right)\\ k\left(L_{n-b+2}^{m},L_{n-b+1}^{m}\right) & \cdots & k\left(L_{n-b+2}^{m},L_{n-1}^{m}\right) & k\left(L_{n-b+2}^{m},L_{n}^{m}\right)\\ \vdots & \ddots & \vdots & \vdots \\ k\left(L_{n}^{m},L_{n-b+1}^{m}\right) & \cdots & k\left(L_{n}^{m},L_{n-1}^{m}\right) & k\left(L_{n}^{m},L_{n}^{m}\right) \end{array}\right]=\left[\begin{array}{cc} K_{fw} & \beta_{L_{n}^{n}}^{T}\\ \beta_{L_{n}^{m}} & \gamma_{L_{n}^{m}} \end{array}\right] \end{array}$$

where $\gamma_{L_{n}^{m}}=k\left(L_{n}^{m},L_{n}^{m}\right)$ is a matrix of size a×a, and $\beta_{L_{n}^{m}}$ is an a×(w−a) matrix, defined by $\beta_{L_{n}^{m}}=\left[k\left(L_{n}^{m},L_{n-b+1}^{m}\right),\cdots ,k\left(L_{n}^{m},L_{n-2}^{m}\right),k\left(L_{n}^{m},L_{n-1}^{m}\right)\right]$. Then, the matrix inversion lemma is repeatedly exploited to calculate $K_{B}^{-1}\left(L\left(n+1\right)\right)$:

(16) $$\begin{array}{l} K_{B}^{-1}\left[L\left(n+1\right)\right]={\left[\begin{array}{cc} K_{fw} & \beta_{L_{n+1}^{m}}^{T}\\ \beta_{L_{n+1}^{m}} & \gamma_{L_{n+1}^{m}} \end{array}\right]}^{-1}=\left[\begin{array}{cc} K_{fw}^{-1}+\lambda {\left(\gamma_{L_{n+1}^{m}}+\beta_{L_{n+1}^{m}}\lambda \right)}^{-1}\lambda^{T} & \lambda {\left(\gamma_{L_{n+1}^{m}}+\beta_{L_{n+1}^{m}}\lambda \right)}^{-1}\\ {\left(\gamma_{L_{n+1}^{m}}+\beta_{L_{n+1}^{m}}\lambda \right)}^{-1}\lambda^{T} & {\left(\gamma_{L_{n+1}^{m}}+\beta_{L_{n+1}^{m}}\lambda \right)}^{-1} \end{array}\right]\\ \lambda =-K_{fw}^{-1}\beta_{L_{n+1}^{m}}^{T} \end{array}.$$

**Figure 3:**
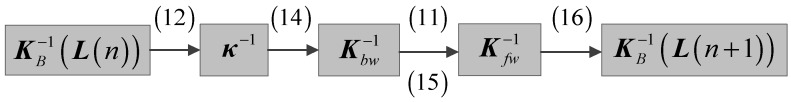
Recursive update process of the kernel Gram matrix inversion.

Taking advantage of Equation (14), $K_{bw}^{-1}$ is obtained by $\kappa^{-1}$, and $K_{B}^{-1}\left(L\left(n+1\right)\right)$ is derived by $K_{fw}^{-1}$ through Equation (16). According to Equations (11) and (15), it can be found that $K_{bw}=K_{fw}$. Above all, as shown in Figure 3, $K_{B}^{-1}\left(L\left(n+1\right)\right)$ can be recursively updated from $K_{B}^{-1}\left(L\left(n\right)\right)$, and thus the calculation of $K_{B}\left(L\left(n+1\right)\right)$ and $K_{B}^{-1}\left(L\left(n+1\right)\right)$ is avoided.

## 3. Description of Hyperspectral Datasets

### 3.1. Synthetic Dataset

**Figure 4:**
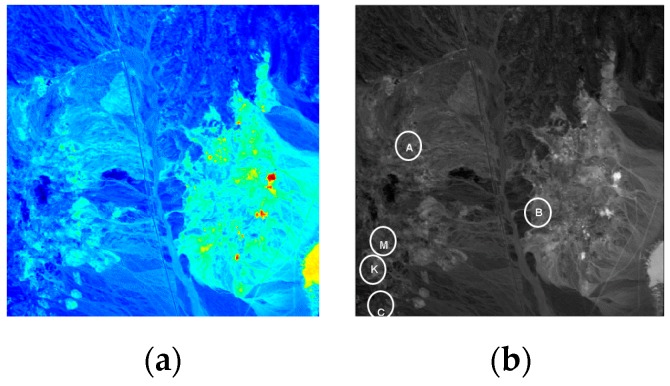
(**a**) Cuprite Airborne Visible/Infrared Imaging Spectrometer sensor (AVIRIS) image; (**b**) Spatial positions of A, B, C, K, and M.

**Figure 5:**
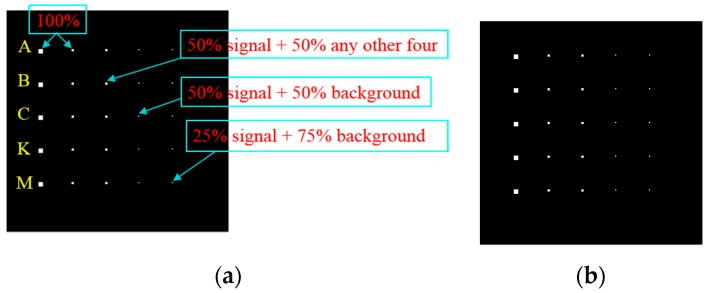
Synthetic dataset. (**a**) Synthetic image; (**b**) Ground truth map.

In Figure 4a, the real Cuprite image, which was collected by the Airborne Visible/Infrared Imaging Spectrometer sensor (AVIRIS) from Nevada in 1997, is used to design the synthetic dataset. It is a 224-band image with a size of 350 × 350 pixels. After removing the water absorption and low SNR bands, only 189 bands are used for the experiments. The five targeted spectral signatures, namely alunite (A), buddingtonite (B), calcite (C), kaolinite (K), and muscovite (M), marked by circles in Figure 4b, are used to simulate 25 anomaly targets, with five panels in each row simulated by the same targeted spectral signature, and each column is superposed by a different proportion of background interference. The size of the panels from the left column to the right column is 4 × 4, 2 × 2, 1 × 1, and 1 × 1, respectively. These 25 panels are then inserted in a synthetic background image with the size of 200 × 200 pixels, as shown in Figure 5a, which is simulated by the sample mean of the real image scene that is corrupted by Gaussian noise to achieve the SNR of 20:1 that is defined in [28]. The ground truth of the synthetic data is presented in Figure 5b.

### 3.2. Pavia Center Dataset

**Figure 6:**
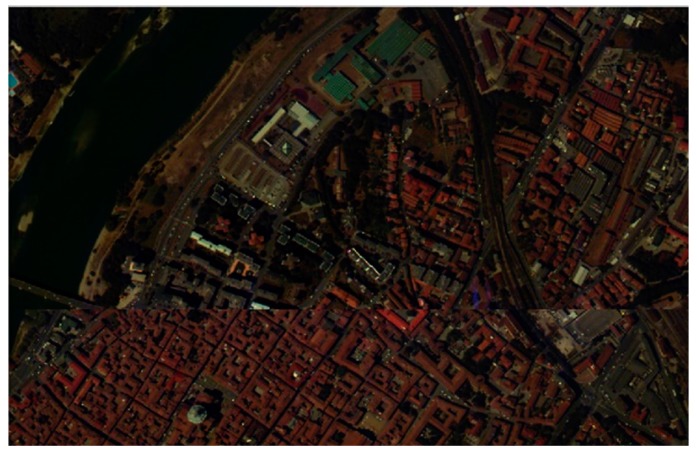
Pavia center hyperspectral image scene.

**Figure 7:**
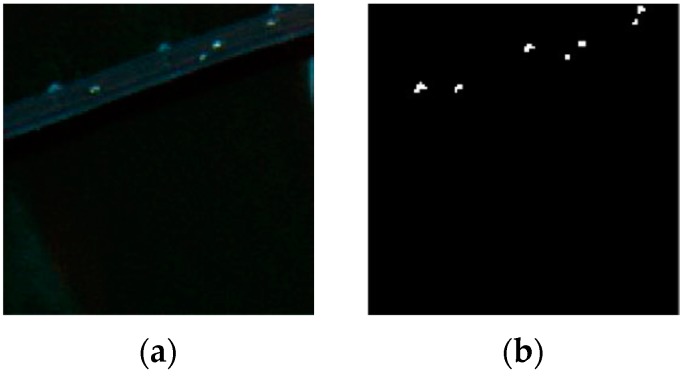
(**a**) Pavia Center dataset; (**b**) Ground truth map.

A Pavia center hyperspectral image scene has been collected by the Reflective Optics System Imaging Spectrometer sensor (ROSIS) from the Pavia city center in northern Italy. As shown in Figure 6, it consists of 115 bands at about 4 nm apart in the spectral region from 0.43 to 0.86 μm with a spatial resolution of 1.3 m. There are 1096 × 715 pixels in the entire image scene. As shown in Figure 7a, we select a subarea of it with the size of 115 × 115 pixels to conduct experiments. A total of 102 spectral bands are used for analysis after removing the atmospheric water bands and low SNR bands. The ground-truth map is displayed in Figure 7b.

### 3.3. San Diego Airport Dataset

**Figure 8:**
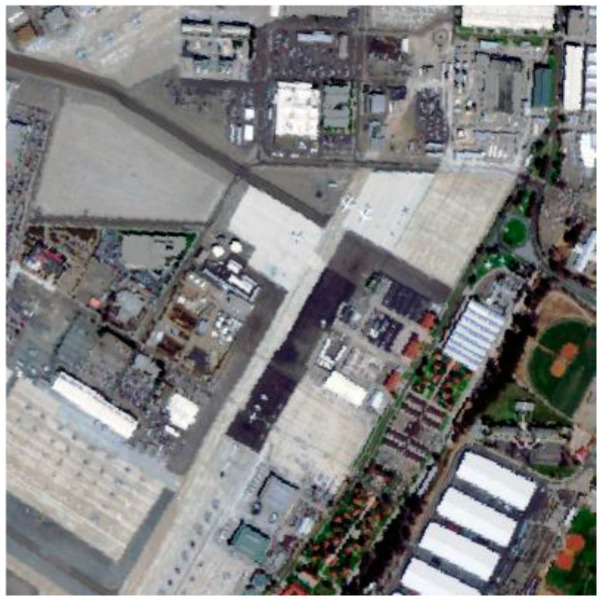
AVIRIS Hyperspectral Image.

**Figure 9:**
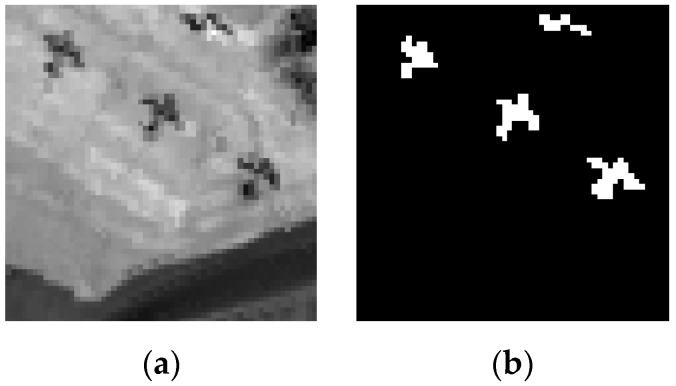
(**a**) San Diego airport dataset; (**b**) Ground truth map.

Figure 8 shows a real hyperspectral image, which was collected by the AVIRIS from San Diego, CA, USA. There are a total of 224 bands ranging from 0.4 to 1.8 μm with a size of 400 × 400 pixels. In this study, atmospheric water bands and low SNR bands were removed, and only the surplus 126 bands are selected for the experiments. The San Diego airport dataset shown in Figure 9a is one subarea of the AVIRIS hyperspectral image. It consists of 60 × 60 pixels with 126 spectral bands for experiment. Figure 9b displays the ground truth map of it.

## 4. Experimental Results and Analysis

In this section, three groups of HSI datasets, including one synthetic image and two real images, are utilized for experiments to demonstrate the performance of the PLP-KRXD.

### 4.1. Optimum Kernel Parameter on the PLP-KRXD

This group of experiments is conducted to find the optimum kernel parameter d on the PLP-KRXD. According to cross-validation, the size of the causal sliding rectangle windows on the synthetic dataset, the Pavia Center dataset, and the San Diego Airport dataset are chosen as (18,5), (15,5), and (12,7), respectively.

The receiver operating characteristic (ROC) [29] curves are employed as one of the frequently used evaluation criteria in anomaly detection. The detection probability $\left(P_{d}\right)$ and false alarm rate $\left(P_{f}\right)$ can be defined as

(17) $$\left\{ \begin{array}{l} P_{d}=\frac{\mathrm{Number}\;\mathrm{of}\;\mathrm{target}\;\mathrm{pixels}\;\mathrm{detected}\;}{\mathrm{Number}\;\mathrm{of}\;\mathrm{total}\;\mathrm{real}\;\mathrm{targets}\;\mathrm{pixels}},\\ P_{f}=\frac{\mathrm{Numbere}\;\mathrm{of}\;\mathrm{background}\;\mathrm{pixels}\;\mathrm{classified}\;\mathrm{as}\;\mathrm{target}}{\mathrm{Number}\;\mathrm{of}\;\mathrm{total}\;\mathrm{pixels}\;\mathrm{in}\;\mathrm{the}\;\mathrm{image}}. \end{array}\right.$$

**Figure 10:**
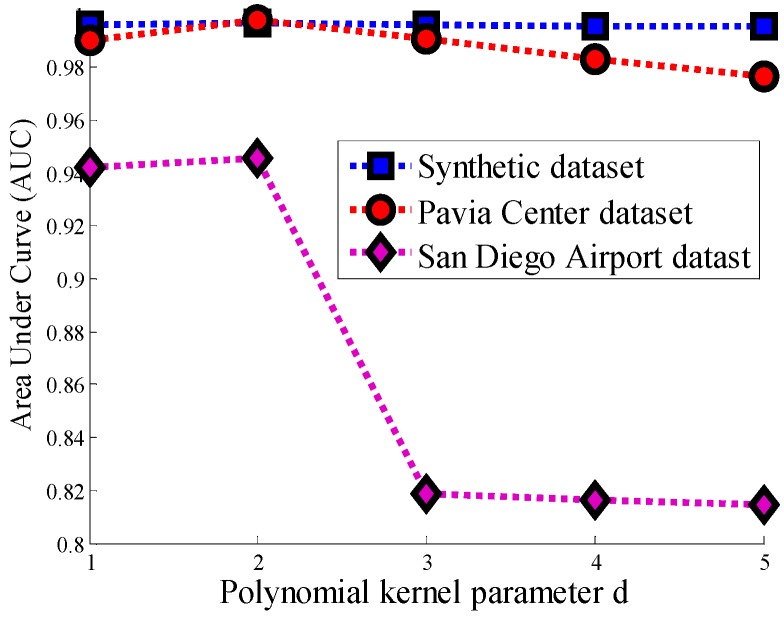
Area under the curve (AUC) of the progressive line processing Kernel-RX detector (PLP-KRXD) with a different polynomial kernel parameter d on three datasets: Synthetic dataset, Pavia Center dataset, and San Diego Airport dataset.

The area under the curve (AUC) is employed as another evaluation criterion to reasonably estimate the accuracy of anomaly detection with respect to the ROC analysis. In detail, a larger AUC value represents a higher accuracy of anomaly detection. Figure 10 illustrates the different AUCs with diverse polynomial kernel parameter d (d=1,2,3,4,5)for the synthetic dataset, the Pavia Center dataset, and the San Diego Airport dataset, respectively. As can be seen in this figure, the AUC of all three HSI datasets reaches a maximum value when d is equal to 2, and then go into a downward trend. For the synthetic dataset and the Pavia Center dataset, the AUC of them has a modest decline, but it declines sharply after reaching the maximum for the San Diego Airport dataset. Hence, the polynomial kernel parameter d is relatively sensitive to the complex hyperspectral dataset. In other words, when the distribution of the data features is simple, the selected d has a weaker effect on the separation of the background and objects.

### 4.2. Effects from the Changing Window Size on the PLP-KRXD

This group of experiments is to investigate that the detection effect of the PLP-KRXD is related to the size of the causal sliding rectangle windows. In these experiments, a and b of the causal sliding rectangle window are separately chosen in a range from 12 to 18 with a step of 3 and 5 to 7 with a step of 1 on the three HSI datasets. By means of cross-validation, the polynomial kernel parameter d of the synthetic dataset, the Pavia Center dataset and the San Diego Airport dataset are all manually set to 2.

Table 1 reveals the output AUC values of the PLP-KRXD for the three HSI datasets with a changing causal sliding rectangle window size. From Table 1, the maximum values obtained in the synthetic dataset, the Pavia Center dataset, and the San Diego Airport dataset are respectively 0.9966, 0.9979, and 0.9458 at the detection windows of (18,5), (15,5), and (12,7). The residuals between the best AUC and the worst AUC of the PLP-KRXD for the three HSI datasets are given in Table 3. Compared to the San Diego Airport dataset, the residuals of the synthetic and Pavia Center datasets are smaller, which means they can weaken the influence resulting from the causal sliding rectangle window size. For the San Diego Airport dataset, due to the distribution of the complex background, its robustness is relatively weaker than the other two HSI datasets.

### 4.3. Detection Performance of PLP-KRXD

In comparison with the KRXD, several experiments are conducted on the three HSI datasets to demonstrate the effectiveness of PLP-KRXD. In these experiments, by cross-validation, the polynomial kernel parameter d for the synthetic dataset, the Pavia Center dataset, and the San Diego Airport dataset are all set to 2. At the same time, the corresponding causal sliding rectangle window size chosen for these three HSI datasets are set to (18,5),(15,5), and (12,7), respectively. For the KRXD, the polynomial kernel parameter d and the dual window size are chosen as 2 and 5/11, respectively.

**Figure 11:**
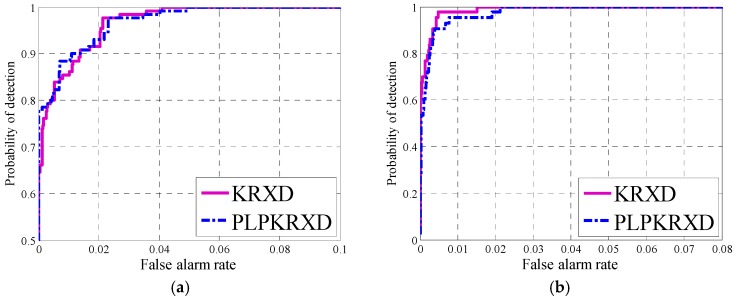

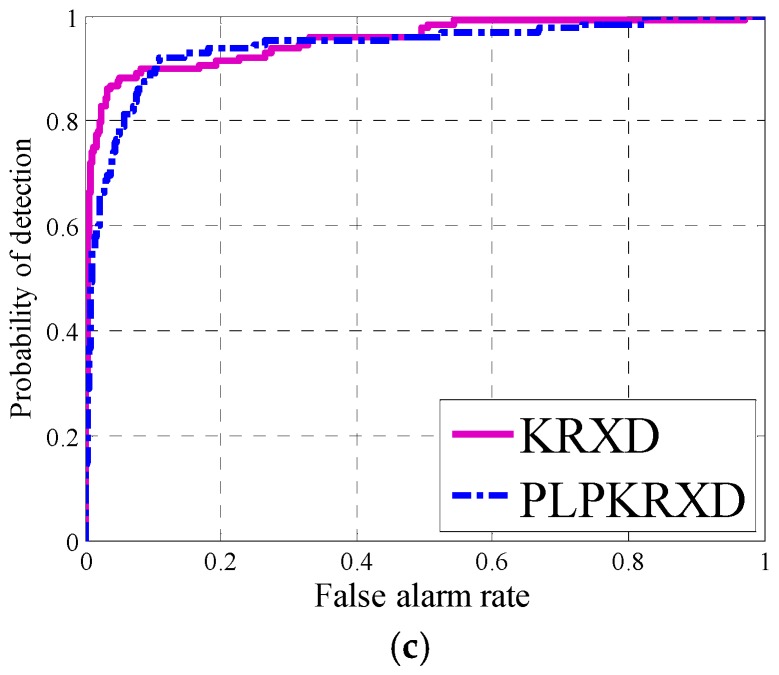
Receiver operation characteristic (ROC) curves obtained by the Kernel-RX detector (KRXD) and the PLP-KRXD on three HSI datasets. (**a**) Synthetic dataset; (**b**) Pavia Center dataset; (**c**) San Diego Airport dataset.

Figure 11 illustrates the ROC curves of the two detectors for the synthetic dataset, the Pavia Center dataset, and the San Diego Airport dataset, respectively. The ROC curves of the KRXD and PLP-KRXD correspond to their best AUC. As can be observed for the synthetic dataset in Figure 11a, the detection precision of the PLP-KRXD is basically the same as that of the KRXD, and it has a higher probability of detection under the lower false alarm probability. For the Pavia Center dataset shown in Figure 11b, the ROC results of the KRXD and the PLP-KRXD are comparable, even if the KRXD is slightly better than the PLP-KRXD. Both of them can achieve a probability of detection of 1 with a lower false alarm rate. For the San Diego Airport dataset shown in Figure 11c, the PLP-KRXD and the KRXD are almost identical in detection accuracy. This result is due to the fact that the recursive process of the PLP-KRXD is derived from the KRXD by applying identity without information leaks. Some subtle distinctions occur between the PLP-KRXD and the KRXD because the KRXD needs to implement the pseudoinverse of the Gram matrix for every pixel, while the PLP-KRXD cancels this operation via the causal sliding rectangle window.

**Figure 12:**
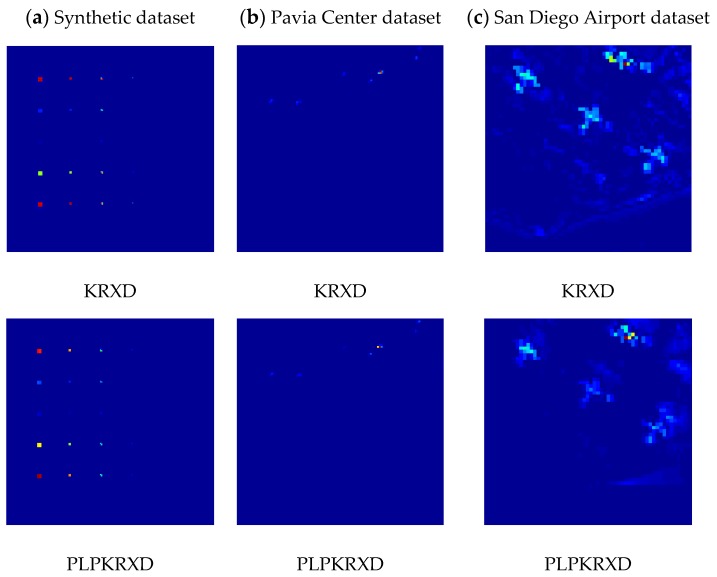
The detection maps of the KRXD and the PLP-KRXD on the three HSI datasets. (**a**) Synthetic dataset; (**b**) Pavia Center dataset; (**c**) San Diego Airport dataset.

**Figure 13:**
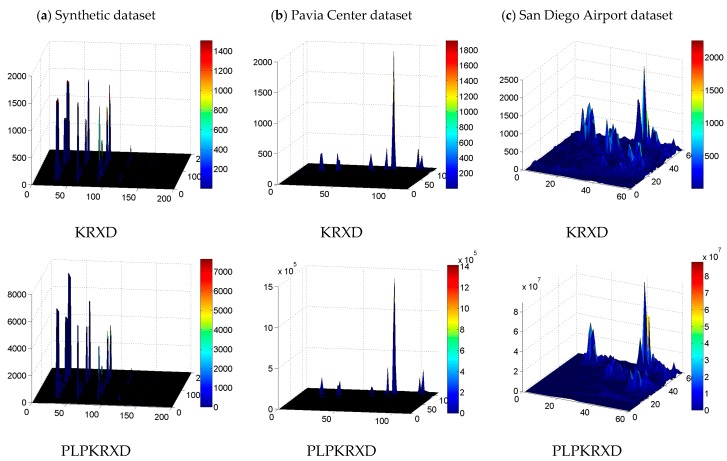
The three-dimensional (3D) plots of the detection results obtained by the KRXD and the PLP-KRXD on three HSI datasets. (**a**) Synthetic dataset; (**b**) Pavia Center dataset; (**c**) San Diego Airport dataset.

Figure 12 presents the detection maps of the KRXD and the PLP-KRXD on the three HSI datasets, respectively. Through comparison, there is no appreciable visual difference between the results of the two anomaly detectors for the synthetic dataset and the Pavia Center dataset. For the San Diego Airport dataset, the detection result obtained by the PLP-KRXD achieves better visual inspection than does the KRXD with regard to the detection maps. In order to make more visualized descriptions for the data and expose more detailed information, Figure 13 illustrates the three-dimensional (3D) plots of the KRXD and the PLP-KRXD on the three HSI datasets. It can clearly be seen that the 3D results of the KRXD and the PLP-KRXD for each group of HSI datasets are extremely comparable; particularly, the PLP-KRXD has better background suppression and accurately detects the anomalies on the San Diego Airport dataset.

**Figure 14:**
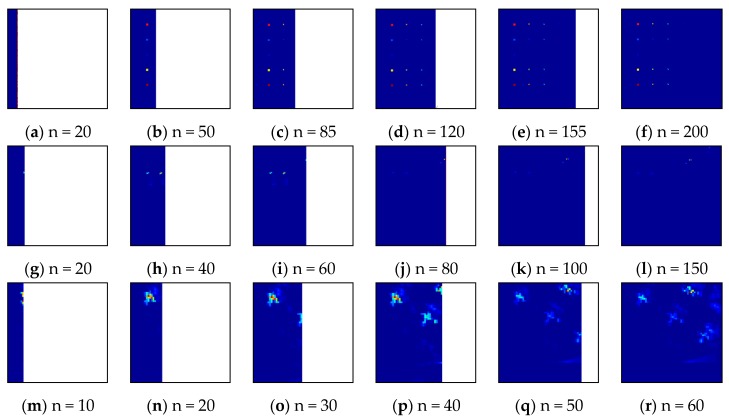
Real-time progressive procedures of PLPKRXD on the three HSI datasets. (**a**–**f**) Synthetic dataset; (**g**–**l**) Pavia Center dataset; (**m**–**r**) San Diego Airport dataset.

Figure 14 provides the line-varying detection maps produced by the PLP-KRXD as it was implemented in real-time on the synthetic dataset, the Pavia Center dataset and the San Diego Airport dataset, respectively. As time progresses, these progressive detection maps are beneficial to visual inspection at varying levels of background suppression. For example, in the Pavia Center dataset, the first three weak anomalies are detected in a better visual assessment. From Figure 14j, k, l, however, as the detection process progresses, these weak anomalies are easily overwhelmed by the subsequent strong anomalies and become dimmer than before. Obviously, this phenomenon cannot be observed using the KRXD that obtains its result in the light of the final detection maps.

### 4.4. Computational Analysis of the PLP-KRXD

Computational analysis is an essential process in real-time processing. Theoretically speaking, the computational complexity is mainly related to the number of multiplication operations. In this paper, it is worth noting that the computational complexity of PLP-KRXD is mainly determined by $K_{B}$ and $K_{B}^{-1}$. Hence, in order to intuitively analyze the time efficiency of PLP-KRXD, Table 2 presents the computational complexity of the kernel Gram matrix and its inversion in both the KRXD and the PLP-KRXD. As can be seen in Table 2, by virtue of recursive processing in the PLP-KRXD, the computational complexity of $K_{B}$ and $K_{B}^{-1}$ in the KRXD is reduced to O(aω⋅J)and $O(a\omega^{2})$ from $O(\omega^{2}\cdot J)$and $O(\omega^{3})$, respectively, where ωrepresents the number of background data sample vectors in the processing window. Note that a must be smaller than ω; hence, the computational complexity of the PLP-KRXD is cheaper than that of the KRXD. In addition, it is evident that the computational complexity is not directly linked to processing time [8]. This is mainly because the PLP-KRXD, using BIL, detects all of the pixels of a line in a one-shot operation while the KRXD, using BIP, processes only one pixel each time.

In order to further perform the concrete comparative analysis and better show the efficiency of the PLP-KRXD, we discuss the total processing time in seconds required by running the KRXD and the PLP-KRXD on the synthetic dataset, the Pavia Center dataset, and the San Diego Airport dataset by averaging five runs, respectively. The computer environments used for experiments are all performed on a 64-bit operating system running on an Intel i7-4770k with a CPU of 3.5 Hz and 16 GB of RAM. As shown in Table 3, compared with the KRXD, the PLP-KRXD using recursive update equations requires far less computing time on the three HSI datasets. It is approximately 40 times faster than the KRXD on the synthetic dataset, and on the San Diego Airport dataset, it is at least 58 times faster.

## 5. Discussion

At the theoretical analysis stage, it is clear that the computation of the current kernel Gram matrix inversion $K_{B}^{-1}\left(L\left(n+1\right)\right)$ needs previous processed information $K_{B}^{-1}\left(L\left(n\right)\right)$ by virtue of Figure 2; therefore, it is necessary to set an initial kernel Gram matrix $K_{initial}^{-1}$. It should be noted that the initial kernel Gram matrix is related to the size of the parallel causal sliding windows. The larger the window size is, the more data sample vectors the initial kernel Gram matrix has. However, a large window size may exceed the time constrains of real-time processing, while small window size easily leads to a singularity in the kernel Gram matrix. It offers a reference for us to determine a proper window size by cross-validation.

In the experiment, the pseudoinverse of the low-rank kernel matrix was used to guarantee the stability of the PLP-KRXD. A common alternative approach for inverting singular and near-singular matrices is to regularize them first [30]. Therefore, in place of the kernel Gram matrix $K_{B}$, we employ a regularization operation $K_{B}^{\prime }=K_{B}+\lambda I$ for some small λ to ensure that all eigenvalues can avoid approaching zero.

According to the analysis of the experiments and simulations, the proposed PLP-KRXD algorithm can maintain the detection accuracy unaltered and accelerate the execution efficiency compared to the KRXD. In each step of deriving the update equations, no information is leaked out, thereby retaining the same detection performance as KRXD while performing real-time processing. The PLP-KRXD recursively updates the kernel Gram matrix and its inversion via a Kalman-like recursive equation, thus avoiding the computation of complex matrices. Besides, parallel causal sliding windows, which only include data samples currently being processed without re-processing previous data samples, are adopted to ensure the causality of real-time processing. Although the PLP-KRXD achieves an over 58-fold speedup, it is sometimes still limited for practical applications. Digital Signal Processors (DSPs) and Graphics Processing Units (GPUs) can be taken into account to speed up real-time processing.

## 6. Conclusions

In this paper, a new real-time anomaly detection of the KRXD algorithm in a BIL fashion is presented. Compared with the KRXD, there are several major advantages that the PLP-KRXD offers. First, the concept of parallel causal sliding windows is developed to ensure the causality of the PLP-KRXD, so that it could be implemented progressively by line in real-time. Second, a new real-time KRXD using BIL via a causal line-varying kernel Gram matrix to detect the anomalies line by line is proposed. Third, weak anomalies can be easily extracted before they are overwhelmed and dominated by the subsequent strong anomaly points, thereby forming data-line varying detection maps. Finally, aiming at the complex computation of the KRXD, this paper takes advantage of the Woodbury matrix identity and the matrix inversion lemma to recursively update the kernel Gram matrix and its inversion to meet the requirement of rapid processing. Through the complexity analysis, for the PLP-KRXD, the computational complexity is significantly reduced and operation efficiency is greatly improved as compared to the KRXD.

Several experiments were conducted on the three HSI datasets to validate the detection performance of the PLP-KRXD algorithm. The experimental results indicate that the PLP-KRXD holds a comparable detection accuracy with the original KRXD and remarkably improves the algorithm’s execution efficiency at the same time.

## Figures and Tables

**Table 1:** AUC performance of the PLP-KRXD with a changing window size for the three hyperspectral imagery (HSI) datasets: Synthetic dataset, Pavia Center dataset, and San Diego Airport dataset.

Windows	Synthetic Dataset	Pavia Center Dataset	San Diego Airport
(12,5)	0.9955	0.9945	0.9412
(15,5)	0.9956	**0.9979**	0.9363
(18,5)	**0.9966**	0.9891	0.9283
(12,6)	0.9956	0.9946	0.9402
(15,6)	0.9886	0.9943	0.9231
(18,6)	0.9964	0.9892	0.9321
(12,7)	0.9955	0.9945	**0.9458**
(15,7)	0.9964	0.9946	0.9426
(18,7)	0.9965	0.9896	0.9413
Residual	0.0080	0.0088	0.0277

**Table 2:** Computational complexity of KRXD and PLP-KRXD.

	KRXD		PLP-KRXD	
	Multiply Operation	Complexity	Multiply Operation	Complexity
$K_{B}$	$\omega^{2}\cdot J$	$O(\omega^{2}\cdot J)$	(ω−a)⋅aJ	O(aω⋅J)
$K_{B}^{-1}$	$\omega^{3}$	$O(\omega^{3})$	$2a\omega^{2}-2a^{2}\omega +a^{3}$	$O(a\omega^{2})$

**Table 3:** Total processing time for three HSI datasets.

	Total Computing Time (seconds)		Speedup
	KRXD	PLPKRXD	
**Synthetic Dataset**	667.671	16.607	**40.204**
**Pavia Center Dataset**	242.698	4.998	**48.559**
**San Diego Airport Dataset**	58.132	1.131	**58.187**
